# Application of two different nasal CPAP levels for the treatment of respiratory distress syndrome in preterm infants—“The OPTTIMMAL-Trial”—Optimizing PEEP To The IMMAture Lungs: study protocol of a randomized controlled trial

**DOI:** 10.1186/s13063-020-04660-0

**Published:** 2020-10-01

**Authors:** Markus Waitz, Corinna Engel, Rolf Schloesser, Ulrich Rochwalsky, Sascha Meyer, Michael Zemlin, Bettina Bohnhorst, Corinna Peter, Marc Hoppenz, Thomas Pabst, Klaus-Peter Zimmer, Axel R. Franz, Harald Ehrhardt, Annesuse Schmidt, Alexander Larsen, Paul Hoffmann, Christoph Haertel, Eric Frieauff, Julia Sandkötter, Katja Masjosthusmann, Philipp Deindl, Dominique Singer

**Affiliations:** 1 Department of General Pediatrics and Neonatology, Center for Child and Adolescent Medicine, Justus-Liebig-University, Feulgenstraße 12, 35392 Gießen, Germany; 2 Center for Pediatric Clinical Studies, University of Tübingen, Frondsbergstraße 23, 72070 Tübingen, Germany; 3 Division of Neonatology, University of Frankfurt, Theodor-Stern-Kai 7, 60590 Frankfurt, Germany; 4 Neonatal Intensive Care Unit, Department of Pediatrics and Neonatology, Saarland University Medical Center, Kirrbergerstrasse 100, 66421 Homburg, Germany; 5 Division of Pediatric Pulmonology, Allergology and Neonatology Hannover Medical School, Carl-Neuberg-Straße 1, 30625 Hannover, Germany; 6 Neonatology and Pediatric Intensive Care Medicine, Hospital Cologne, Amsterdamer Strasse 59, 50735 Köln, Germany; 7 Department of Neonatology and Center for Pediatric Clinical Studies, University of Tübingen, Calwerstr. 7, 72076 Tübingen, Germany; 8 German Center for Lung Research (DZL), Universities of Giessen and Marburg Lung Center (UGMLC), Feulgenstraße 12, 35392 Gießen, Germany; 9 University Children’s Hospital, University of Würzburg, Josef-Schneider-Str. 2, 97080 Wuerzburg, Germany; 10 Department of General Pediatrics, University Children’s Hospital Muenster, Albert-Schweitzer-Campus 1, 48149 Muenster, Germany; 11 Division of Neonatology and Pediatric Intensive Care, Department of Pediatrics, University Medical Center Hamburg-Eppendorf, Martinistrasse 52, 20246 Hamburg, Germany

**Keywords:** Nasal CPAP, RDS, Preterm infants, PEEP

## Abstract

**Background:**

Nasal continuous positive airway pressure (CPAP) applies positive end-expiratory pressure (PEEP) and has been shown to reduce the need for intubation and invasive mechanical ventilation in very low birth weight infants with respiratory distress syndrome. However, CPAP failure rates of 50% are reported in large randomized controlled trials. A possible explanation for these failure rates is the application of insufficient low levels of PEEP during nasal CPAP treatment to maintain adequate functional residual capacity shortly after birth. The optimum PEEP level to treat symptoms of respiratory distress in very low birth weight infants has not been assessed in clinical studies. The aim of the study is to compare two different PEEP levels during nasal CPAP treatment in preterm infants.

**Methods:**

In this randomized multicenter trial, 216 preterm infants born at 26 + 0–29 + 6 gestational weeks will be allocated to receive a higher (6–8 cmH_2_O) or a lower (3–5 cmH_2_O) PEEP during neonatal resuscitation and the first 120 h of life. The PEEP level within each group will be titrated throughout the intervention based on the FiO_2_ (fraction of inspired oxygen concentration) requirements to keep oxygenation within the target range. The primary outcome is defined as the need for intubation and mechanical ventilation for > 1 h or being not ventilated but reaching one of the two pre-defined CPAP failure criteria (FiO_2_ > 0.5 for > 1 h or pCO_2_ ≥ 70 mmHg in two consecutive blood gas analyses at least 2 h apart).

**Discussion:**

Based on available data from the literature, the optimum level of PEEP that most effectively treats respiratory distress syndrome in preterm infants is unknown, since the majority of large clinical trials applied a wide range of PEEP levels (4–8 cmH_2_O). The rationale for our study hypothesis is that the early application of a higher PEEP level will more effectively counteract the collapsing properties of the immature and surfactant-deficient lungs and that the level of inspired oxygen may serve as a surrogate marker to guide PEEP titration. Finding the optimum noninvasive continuous distending pressure during early nasal CPAP is required to improve CPAP efficacy and as a consequence to reduce the exposure to ventilator-induced lung injury and the incidence of chronic lung disease in this vulnerable population of very preterm infants.

**Trial registration:**

drks.de DRKS00019940. Registered on March 13, 2020

## Administrative information

**Table:** 

Title {1}	Use of Two Different PEEP levels During Resuscitation and Treatment of RDS in preterm infants – a randomized controlled multicenter trial. “OPTTIMMAL-Trial” – Optimizing PEEP To The IMMAture Lungs
Trial registration {2a and 2b}.	2a The trial has been registered at the DRKS (German Clinical Trial Register). 2b The register collects all items from the World Health Organization Trial Registration Data Set.
Protocol version {3}	Version 2.0, December 04th, 2019
Funding {4}	The study is funded by the Else- Kroener Fresenius Stiftung, Bad Homburg, Germany. Types of financial support: Patient Management Fee, Staff Funding, Patient Insurance
Author details {5a}	1 Department of General Pediatrics and Neonatology, Justus-Liebig-University of Giessen, Germany. 2 Department of Neonatology, University Children’s Hospital Tuebingen, Germany. 3 Center for Pediatric Clinical Studies, University Children’s Hospital Tuebingen, Germany 4 Division of Neonatology, University of Frankfurt, Germany 5 Neonatal Intensive Care Unit, Department of Pediatrics and Neonatology, University Children’s Hospital of Saarland, Germany 6 Division of Pediatric Pulmonology, Allergology and Neonatology, Hannover Medical School, Germany. 7 Department of Neonatology and Pediatric Intensive Care Medicine, Children’s Hospital, Cologne, Germany. 8 German Center for Lung Research (DZL), Universities of Giessen and Marburg Lunge Center (UGMLC), Giessen, Germany.
Name and contact information for the trial sponsor {5b}	Else-Kröner-Fresenius Foundation Street: Am Pilgerrain 15 Postal zip code 61352 City: Bad Homburg Country: Germany Email: kontakt@ekfs.de
Role of sponsor {5c}	The study funder is not involved nor has any responsibility for the collection, management, analysis, and interpretation of data; writing of the report; decision to submit the report for publication. They will not have ultimate authority over any of these activities.

## Introduction

### Background and rationale {6a}

Very low birth weight infants (VLBWI) with surfactant deficient lungs frequently develop respiratory distress syndrome (RDS) with the need of early respiratory support in the delivery room and during the active phase of RDS (i.e., first 5 days of life) [1]. The use of nasal CPAP to avoid intubation and the consecutive detrimental effects of invasive mechanical ventilation to the immature lungs has been shown to reduce the rate of bronchopulmonary dysplasia (BPD) and death in meta-analyses [2]. The diagnosis of BPD is associated with lifelong impaired lung function and negatively affects the neurodevelopmental outcome in this high risk group of infants [3]. Nasal CPAP in the delivery room is now the recommended primary respiratory support in preterm infants with RDS. Results of large randomized controlled trials comparing early nasal CPAP versus primary intubation found that approximately 46.0–51.2% of infants can be saved from intubation with this approach [4, 5]. However, almost half of the infants in the large CPAP trials failed on CPAP, which ultimately resulted in exposure to some degree of invasive mechanical ventilation and may explain limited effects on the reduction of the incidence of BPD [1, 6, 7]. Application of early nasal CPAP aims to recruit the lungs and maintain functional residual capacity (FRC). The degree of lung recruitment is influenced by the level of PEEP applied to the immature lung [8]. Expert panels from international societies currently recommend a PEEP level of 5–6 cmH_2_O [4, 9]. However, as the authors state, evidence for these recommendations is very limited. No clinical studies specifically assessed the effect of different PEEP levels during the active phase of RDS in preterm infants on outcomes such as the need for invasive mechanical ventilation and other neonatal morbidities (i.e., BPD). Results of a secondary analysis from a cohort study in 34 international centers that participated in a nasal intermittent positive pressure ventilation trial indicate a large variation of PEEP levels used in clinical practice during neonatal resuscitation and the first 28 days of life (i.e., 3–9 cmH_2_O) [10]. Only one clinical trial with a small sample size, including preterm infants beyond the active phase of RDS, randomized subjects to receive a higher or a lower level of PEEP after extubation (4–6 cmH_2_O versus 7–9 cmH_2_O) and found a significantly lower rate of reintubation in the higher PEEP group [11]. Results from animal studies further suggest that using higher PEEP levels (8–12 cmH_2_O) improves gas exchange and avoids lung collapse when compared to lower levels of PEEP [12, 13]. The optimum level of PEEP during nasal CPAP that effectively maintains FRC and avoids mechanical ventilation in preterm infants with RDS is unknown and remains to be determined.

The proposed randomized controlled trial was designed and powered to compare the effect of two different PEEP levels during nasal CPAP (6–8 cmH_2_O versus 3–5 cmH_2_O) in preterm infants in the first 120 h of life on the need for intubation and the incidence of pre-defined CPAP failure criteria.

### Objectives {7}

The primary hypothesis of this study is that the use of a higher PEEP range in preterm infants born at 26 + 0–29 + 6 weeks gestational age (GA) receiving prophylactic nasal CPAP support after birth reduces the incidence of intubation and/or meeting predefined CPAP failure criteria within the first 120 h of life when compared to the application of a lower PEEP range.

### Trial design {8}

This will be an unblinded multicenter randomized controlled parallel group comparison of two different PEEP ranges during nasal CPAP support in the first 120 h of life.

## Methods: participants, interventions, and outcomes

### Study setting {9}

#### Participating centers

The below listed centers and site investigators will be actively recruiting for the trial:

- Department of General Pediatrics and Neonatology, Justus-Liebig-University of Giessen; Harald Ehrhardt, Markus Waitz.
- Department of Neonatology, University of Tuebingen; Axel Franz.
- Division of Neonatology, University of Frankfurt; Rolf Schloesser, Ulrich Rochwalsky.
- Department of Pediatrics and Neonatology, University Children’s Hospital of Saarland; Sascha Meyer, Michael Zemlin.
- Division of Pediatric Pulmonology, Allergology and Neonatology, Hannover Medical School; Bettina Bohnhorst, Corinna Peter.
- Department of Neonatology and Pediatric Intensive Care Medicine, Children’s Hospital, Cologne; Marc Hoppenz, Thomas Pabst.

### Eligibility criteria {10}

#### Inclusion criteria

- GA at birth 26 + 0/7 to 29 + 6/7 weeks

#### Exclusion criteria

- Severe congenital anomalies affecting breathing control (cerebral anomalies, chromosomal anomalies, prenatally diagnosed intracranial hemorrhage) *or* gas exchange (pulmonary hypoplasia due to congenital diaphragmatic hernia or oligo/anhydramnios present at < 22 weeks GA or other pulmonary or intrathoracic malformations, etc.) *or* hemodynamics (cyanotic heart disease, ductal dependent systemic perfusion, or similar)
- Decision not to provide full life support/decision for palliative care only before study entry
- Parents not able to understand the study due to language barriers

### Who will take informed consent? {26a}

All pregnant women who are admitted to the local prenatal/maternity wards with threatening preterm delivery before 29 + 6/7 weeks should be approached. Both parents will be asked for consent for their baby to participate in the study given that it will be delivered between 26 + 0 and 29 + 6 weeks of gestation. Informed consent will be taken by good clinical practice (GCP) qualified staff members.

### Additional consent provisions for collection and use of participant data and biological specimens {26b}

To date, no sampling of biological material is planned during the study.

## Interventions

### Explanation for the choice of comparators {6b}

Since the optimum level of PEEP to treat RDS is unknown, we decided to choose the levels of PEEP within the reported PEEP levels used in clinical practice from large surveillance data (3–9 cmH_2_O) and within the limits of current treatment guidelines [4, 9, 10]. To allow for effective discrimination between the two treatment groups, we decided that the difference of the applied level of PEEP in the delivery room must be exactly 3 cmH_2_O. At last, the choice of the defined PEEP levels yielded the highest agreement in the pre-study meetings of all participating centers.

### Intervention description {11a}

#### Study intervention

The study intervention is the application of early nasal CPAP/nasal intermittent positive pressure ventilation (NIPPV) with higher PEEP range (6–8 cmH_2_O, intervention group) compared to a lower PEEP range (3–5 cmH_2_O, control group) in the first 120 h of life.

#### Interventional period

The intervention starts immediately after birth with the first application of nasal CPAP/NIPPV in the delivery room. The study intervention ends at a postnatal age of 120 h. Randomized subjects will receive nasal CPAP/NIPPV immediately after birth using a short nasopharyngeal tube, face mask, or binasal prongs (center specific). The PEEP level will be set within the allocated PEEP range: PEEP_low_ (3–5 cmH_2_O) or PEEP_high_ (6–8 cmH_2_O). During resuscitation, the *initial* PEEP setting in the intervention group must be set 3 cmH_2_O higher than in the control group and will be determined by center guideline prior to study start. The initial PEEP will be *maintained* during the delivery room management and will not be adjusted. Application of PEEP in the delivery room will be provided by a T-Piece resuscitator or a conventional ventilator. The use of a self or flow-inflating bag is not permitted for initial respiratory support. Subjects will be further resuscitated according to the European Consensus Guidelines [4]. Sustained inflation maneuvers can be applied according to local policies for initial stabilization. Primary nasal NIPPV (synchronized or not) can be used in the delivery room. After admission to the neonatal intensive care unit, the PEEP level will be adjusted according to the level of inspired oxygen required to meet the center-specific study SpO_2_ target range within the maximum range of 85–95% throughout the interventional period using the following PEEP titration protocol: (PEEP adjustments should be made on a half-hourly basis to allow the assessment of effects).

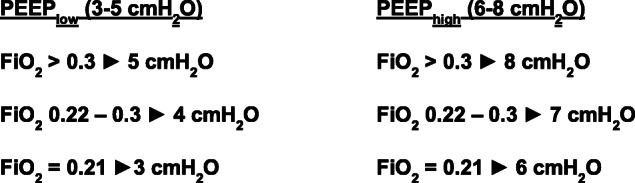



Prior to study initiation, we strongly recommend local guidelines for intubation and mechanical ventilation be defined, as well as the indication criteria for surfactant administration that have to be applied equally within both PEEP groups. Suggested intubation criteria within the study are:

FiO_2_ ≥ 0.5 for > 1 h and/or pCO_2_ ≥ 70 mmHg in two consecutive blood gas analysis > 2 h apart after the application of a maximum of 2 surfactant doses (either INSURE (intubate surfactant extubate) or less invasive, i.e., LISA).

If a study subject requires intubation, decisions on the mode of invasive ventilation, weaning strategies, and extubation are at the discretion of the responsible medical team of the study site with settings and modes identical for both treatment groups. After extubation, the PEEP will be set and adjusted according to the initially assigned PEEP range during nasal CPAP/NIPPV. After the study intervention period, the medical team at each study site will decide on the further mode of respiratory support and weaning strategies.

#### Continuous documentation of the study intervention

In both treatment groups, FiO_2_, set PEEP levels, and pressures during CPAP and NIPPV (mean airway pressure, peak inspiratory pressure, inspiratory time) will be recorded every hour throughout the interventional period on a worksheet and has to be signed by a study physician. All data will be transferred from hardcopy to electronic CRF files and are stored within the database of the center for pediatric clinical studies (CPCS), University of Tuebingen. This database fulfills all GCP and FDA CFR part 11 requirements. After termination of the study, all datasets are backed up within a locked area at the Justus-Liebig-University of Giessen and at the Eberhard-Karls-University of Tuebingen for at least 10 years after the end of the study.

### Criteria for discontinuing or modifying allocated interventions {11b}

#### Individual preterm end of study intervention

##### For the individual patient

Treatment may be terminated for safety concerns by the attending physician together with the local principal investigator or according to the wish of the patient’s parents or legal representatives at any time. The reasons for premature termination of the study intervention have to be documented in the study database. These patients will be maintained in the study, study visits have to be done as planned, and their outcome will be included in the intention-to-treat analysis.

#### Preterm end of study

##### For a study center

A study center not following the protocol or failing to recruit patients may be closed prematurely. All infants already recruited at that center will be maintained in the study, study visits have to be done as planned, and their outcome will be included in the intention-to-treat analysis.

##### For the whole study

For safety: all potential complications related to the intervention occurring during the care of an infant enrolled in this trial have to be reported immediately to the coordinating investigator according to ICH-GCP guidelines and national and European regulations. Furthermore, information on all prematurity-associated complications is collected in the study database. These will include safety reports after 50, 100, and 150 completely documented patients to the data monitoring committee (DMC), who will continuously keep track of the incidence of such events in both study groups. The trial will be stopped by the coordinating investigator on the advice of the DMC, if the risk-benefit ratio of the study intervention (i.e., application of higher PEEP ranges) is significantly changed based on new published data becoming available. The trial will also be stopped temporarily or permanently in case that safety outcomes (i.e., mortality or major diseases of prematurity) occur more frequently in the treatment group or if complications directly related to the study intervention (e.g., adverse events directly attributable to device failures) require a change in the risk-benefit assessment.

### Strategies to improve adherence to interventions {11c}

Local principal investigators and team members are required to participate in a pre-study meeting where details of study protocol, data collection, the application, and adjustments of PEEP will be discussed. All participating centers will receive detailed written instructions. In cases of uncertainty, it will be possible to contact the Center for Pediatric Clinical Studies (CPCS, University of Tuebingen) at any time.

### Relevant concomitant care permitted or prohibited during the trial {11d}

#### Management with alternative noninvasive respiratory support (i.e., NIPPV)

NIPPV is frequently used as primary mode of noninvasive ventilation in the delivery room and the first days of life. Because the settings during NIPPV affect the applied mean airway pressure and gas exchange, NIPPV settings and weaning during the study intervention must be equally used in terms of inspiratory times, respiratory rate, and peak inspiratory pressures, which will be monitored and documented during the study intervention. We strongly recommend a center-specific protocol for the use of NIPPV prior to study initiation.

#### Caffeine therapy

Randomized infants will receive a loading dose of caffeine citrate of (10)–20 mg/kg and a maintenance dose of (5)–10 mg/kg/days. Up to 20 mg/kg/days is permitted to prevent intubation due to apnea of prematurity. Because the optimal dose of caffeine has not yet been established, exact dosing will be left to the standards of the participating centers—but centers will need to have a written dosing scheme. Furthermore, caffeine treatment will be monitored throughout the study to detect any bias in caffeine use early.

### Provisions for post-trial care {30}

Patients that are enrolled into the study are covered by patient insurance (HDI Global SE).

### Outcomes {12}

The primary outcome is meeting one of the following treatment failure criteria within the first 120 h after birth:

1. Need for intubation and invasive mechanical ventilation for > 1 h
  or
2. FiO_2_ ≥ 50% for > 1 h after NICU admission
  or
3. pCO_2_ ≥ 70 mmHg in two consecutive blood gas analyses > 2 h apart

Secondary outcomes are:

1. Rates of invasive or less invasive surfactant administration
2. Cumulative dose of surfactant in mg/kg birth weight
3. Rate of intubation and invasive mechanical ventilation within the first 120 h after birth
4. Duration of invasive mechanical ventilation within the first 120 h of life (cumulative hours)
5. Duration of invasive mechanical ventilation until PMA36 or death (cumulative hours)
6. Duration of any respiratory support until PMA36 or death (cumulative days)
7. Rate and severity distribution of BPD
8. Rate of discharge with supplemental oxygen
9. Rates of postnatal systemic dexamethasone and/or hydrocortisone intended for chronic lung disease
10. Rate of inhaled steroids
11. Need for inotropes/vasopressors within the first 120 h of life
12. Need for steroids for treatment of arterial hypotension within the first 120 h of life
13. Mean FiO_2_ requirements within the first 120 h of life
14. Mean airway pressure within the first 120 h after birth

Further outcomes are:

1. Bayley Scales of Infant Development (3rd) Edition at 24 months corrected age
2. Visual assessment at 24 months corrected age
3. Hearing assessment at 24 months corrected age
4. GMFCS-Score at 24 months corrected age
5. Wechsler preschool and primary scale of intelligence (WPPSI-III) at 60 months corrected age
6. Lung function at 60 months corrected age
7. MRI of the lung and brain at 60 months

### Participant timeline {13}

#### Study procedures, examination methods, and outcome assessment

**Fig. 1:**
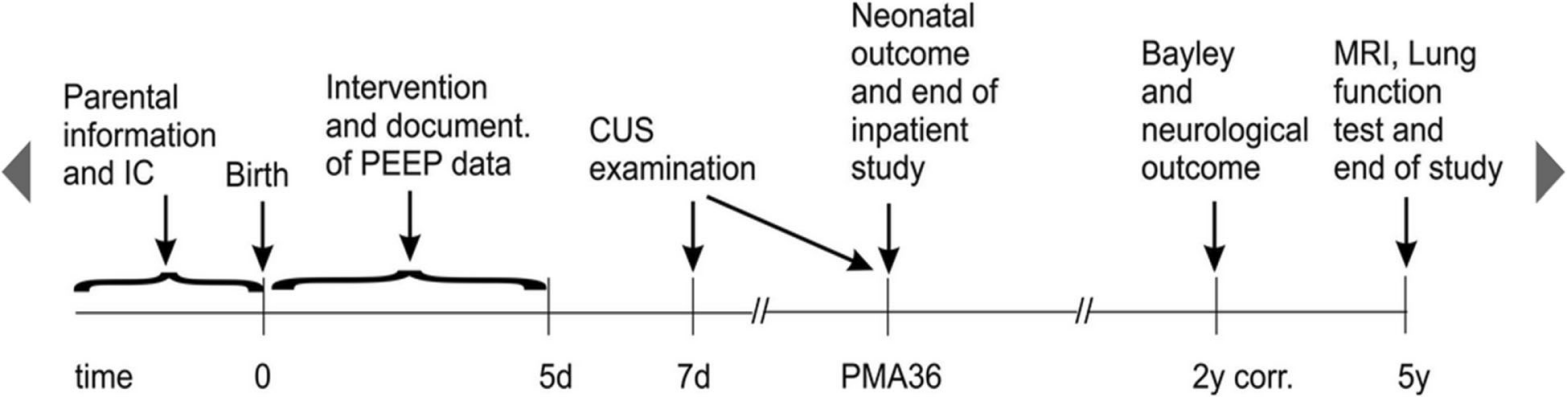
Overview about the study procedures and examinations. IC, informed consent; CUS, cerebral ultrasound; MRI, magnetic resonance imaging; PMA, postmenstrual age

**Fig. 2:**
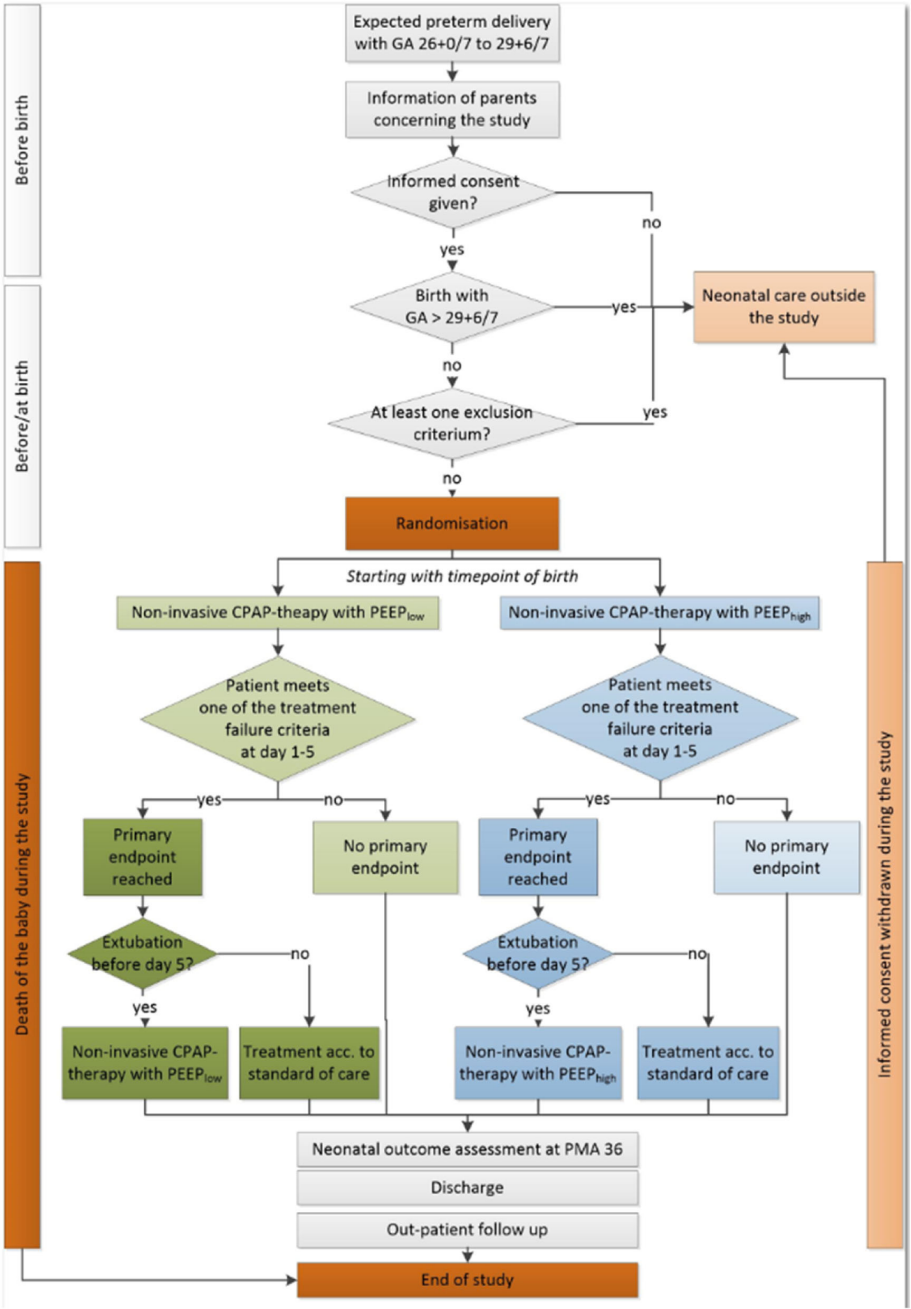
Study process for the individual patient

The description of the study procedures and examinations are shown in Fig. 1. A summary of important outcomes and time points of assessment are shown in Table 1. A detailed description of the study process for the individual patient is summarized in Fig. 2.

**Table 1:** Time points of interventions and outcome assessment of outcomes during the study

Points of action	Screening	Baseline	Visits			End of inpatient study	Follow-up		End of study
Visit	1	2	3	4	5	6	7	8	9
Time point/age	Prior birth	0 h	120 h	Day 7	PMA 36	Discharge	24 months corrected age	60 months of age	
Inclusion criteria	●								
Exclusion criteria	●								
Informed consent	●								
**Neonatal and maternal characteristics**									
Data		●							
**Study intervention/ventilation**									
Start		●							
End			●						
PEEP data			●						
Surfactant treatment			●						
Ventilation data			●		●	●			
**Safety**									
Serious adverse events	Continuous reporting until discharge								
**Outcome assessment**									
BPD					●				
Death					●				●
ROP					●				
NEC					●				
FIP					●				
PDA					●				
Nosocomial infections					●				
Brain injury (CUS)				●	●				
Feeding data					●				
Inotropes					●				
Postnatal steroids						●			
Bayley III/GMFCS							●		
Visual and hearing							●		
Lung function								●	
WPPSI-III								●	
Lung/brain MRI								●	
Parental questionnaire	Every 3 months after discharge								

*BPD* bronchopulmonary dysplasia, *CUS* cerebral ultrasound, *FIP* focal intestinal perforation, *GMFCS* gross motor function classification scale, *MRI* magnetic resonance imaging, *NEC* necrotizing enterocolitis, *PDA* persistent ductus arteriosus, *PMA* postmenstrual age, *ROP* retinopathy of prematurity

### Sample size {14}

Based on a pre-study survey among participating centers and the results of the AMV study [14], we expect 50% primary endpoints within the control group (lower PEEP level). The intervention will be regarded as being successful if it lowers the rate of the primary endpoints to 30%. This follows the expectations within similar studies [15]. To lower the rate of primary endpoints from 50 to 30% and to confirm this via Cochran-Mantel-Haenszel test (level of significance = 0.05, power = 80%), a sample size of 103 patients per group will be needed. To account for 5% drop outs, an overall sample size of 216 patients have to be recruited and treated within the study.

### Recruitment {15}

#### Recruitment and study duration

Patients will be recruited in 6 German tertiary care neonatal centers. Results of a systematic surveillance protocol prior to study start yielded a number of 240 eligible preterm infants meeting the inclusion criteria annually in all participating centers. Based on the assumption that approximately 50% of eligible infants will be included in the study, the recruitment period is expected to be 27 months. All preterm infants who are born at the participating institutions with a gestational age at birth of 26 + 0/7 to 29 + 6/7 weeks have to be screened for the study. Each screened patient is given a patient screening number (PSN) in consecutive order according to the screening log in the investigator site file (ISF). This number is the overall identifier of the pseudonymized patient throughout the study. For every screened patient, a screening form has to be filled in the electronic case report form (eCRF). This enables the documentation of non-biased recruitment. The screening eCRF form will document presence and absence of inclusion and exclusion criteria, whether the parents had been approached and whether informed consent was given, but will not document any patient identifiers. Outborn infants will not be screened as study treatment has to start immediately after birth. Patients can only be enrolled into the study if informed written consent was given by both parents/guardians (by the only parent/guardian in case of single-parent/guardian families) before birth. In case parents are less than 18 years of age, the relevant legal guardian(s) of the child has/have to sign the informed consent. Following multiple birth, parents are asked whether the infants could be randomized separately. If parents wish randomization to one study arm, the second child is treated like the randomized child, but not recruited to the study. The parental wish is documented on the informed consent form.

## Assignment of interventions: allocation

### Sequence generation {16a}

#### Randomization and allocation concealment

Infants will be randomly assigned in a 1:1 ratio with variable block sizes. Randomization lists will be stratified by center and gestational age (26 + 0–27 + 6 and 28 + 0–29 + 6 weeks). Sequentially numbered, sealed, opaque envelopes indicating the study group will be prepared. Provision of randomization envelopes will be done by an independent institution not involved in patient recruitment.

### Concealment mechanism {16b}

Please see the “Sequence generation {16a}” section.

#### Implementation {16c}

Please see the “Sequence generation {16a}”section.

### Assignment of interventions: blinding

#### Who will be blinded {17a}

Blinding of doctors, nurses, and parents for the intervention is not possible in this study.

#### Procedure for unblinding if needed {17b}

Please see the “Who will be blinded {17a}” section.

## Data collection and management

### Data management plan, quality assurance, and monitoring

A data management plan will be written describing the whole flow of the study data. Documentation of study data will be done by the local principal investigator via eCRF within a web-based study database. Source data will remain within the study sites. Quality and correctness of the study data will be ascertained by GCP-conform monitoring of the study. Monitoring will be done on a risk-adapted basis and will include remote and on-site monitoring of the data by trained monitors.

### Plans for assessment and collection of outcomes {18a}

Please see the “Study procedures, examination methods, and outcome assessment” section.

### Plans to promote participant retention and complete follow-up {18b}

#### Data management {19}

Please see the “Data management plan, quality assurance, and monitoring” section.

#### Confidentiality {27}

##### Plans for collection, laboratory evaluation, and storage of biological specimens for genetic or molecular analysis in this trial/future use {33}

To date, no sampling of biological material is planned during the study.

## Statistical methods

### Statistical analysis {20a}

The following analysis populations are defined within this study:

1. *Intention to treat population*
  Consists of all patients included into the study of whom written parental consent was obtained and not withdrawn.
2. *Safety population*
  The safety population is identical to the intention to treat population and consists also of all patients included into the study of whom written parental consent was obtained and not withdrawn.
3. *Per protocol population*
  Patients fulfilling at least one of the following criteria will be excluded from the per protocol-population:
  PEEP level is not documented or not between 3 and 5 cmH_2_O—if the infant is in the low PEEP group—or 6–8 cmH_2_O—if it is randomized in the high PEEP group—for more than 10% of time during the intervention period.
  Intubation within the first 10 min after birth and invasive mechanical ventilation for more than 1 h.

The analysis of the primary endpoint will be done by Cochran-Mantel-Haenszel test stratified for treatment groups. The primary endpoint will be re-evaluated by Cochran-Mantel-Haenszel test adjusted for gestational age group (26 + 0–27 + 6 versus 28 + 0–29 + 6 weeks) and center. If the final number of centers does not allow adjustment for single centers, centers will be grouped. The grouping of centers will be justified within the statistical analysis plan. All secondary endpoints will be compared by Cochran-Mantel-Haenszel test or Student’s *t* test. In case of non-normally distributed data, Student’s *t* test will be replaced by Wilcoxon-Mann-Whitney test. The distribution of severity of BPD will be compared between the treatment groups by generalized logit model. Clinical and demographic characteristics of maternal and neonatal data will be described by mean and standard deviation, median, quartiles and minimum/maximum or rate and percentage. The analysis of the follow-up endpoints will be specified in an amendment to the study protocol before start of the 2- and 5-year follow-up. In case of more than 10% missing values concerning the primary endpoint, a worst case/best case analysis for this endpoint will be performed in the intention to treat population as sensitivity analyses and results will be included into the final report. No sensitivity analyses will be done for secondary or further relevant endpoints. In case of more than 20% missing values concerning one of the endpoints until PMA36, this endpoint will only be tabulated but no statistical test will be performed due to risk of biased data.

All analyses will be predefined in a statistical analysis plan written before end of study and completion of data monitoring. Only the analysis of the primary outcome variable in the intention-to-treat population will be considered confirmatory on a level of significance of 0.05. All other analyses including the analysis of the primary outcome variable in the per protocol population and all analyses concerning secondary endpoints will be considered exploratory only.

### Interim analyses {21b}

Not planned

### Methods for additional analyses (e.g., subgroup analyses) {20b}

There are no subgroup analyses predefined within the study protocol. If appropriate, they will be defined within the statistical analyses plan.

### Methods in analysis to handle protocol non-adherence and any statistical methods to handle missing data {20c}

Please see the “Statistical analysis {20a}” section.

### Plans to give access to the full protocol, participant level-data, and statistical code {31c}

Access to the full study protocol and the final statistical analysis plan will be given upon request. There is no plan to give access to participant-level data for data protection reasons.

## Oversight and monitoring

### Composition of the coordinating center and trial steering committee {5d}

Please see the “Composition of the data monitoring committee, its role and reporting structure {21a}” section.

### Composition of the data monitoring committee, its role and reporting structure {21a}

The DMC is represented by two neonatologists independent from the sponsor or competing interests. The DMC will be contacted by the two coordinating investigators (Harald Ehrhardt and Markus Waitz) based on their expertise (being at least principal investigators in clinical trials for more than 5 years in the past and experts in the field of neonatology). Details (name and contact information) of the composition of the DMC are provided in the study protocol or upon request. Safety aspects will be documented continuously throughout the study including the categories expected serious adverse events (eSAE) and unexpected serious adverse events (uSAE) events and will be reported within safety reports to the DMC. Safety analyses will be done after 50, 100, and 150 included patients have reached PMA 36 weeks. Reports of these data will be sent to the DMC members by the responsible biometrician (CPCS, University of Tuebingen). Based on the reported data, the DMC will provide recommendations on continuation of the trial and will report its decision to the coordinating investigators.

### Adverse event reporting and harms {22}

The following expected serious (eSAE) and unexpected serious (uSAE) adverse events known to occur in this population of very preterm infants will be reported to the DMC as rates and percent, stratified for treatment group.

*eSAE:*

- Death before discharge
- Necrotizing enterocolitis stage ≥ 2 or need for surgery
- Focal intestinal perforation
- Any intraventricular hemorrhage
- Any intraventricular hemorrhage ≥ 3°
- Periventricular leucomalacia
- Any persistent ductus arteriosus requiring treatment (medical/surgical)
- Any retinopathy of prematurity requiring treatment
- Rate of air leak syndromes (i.e., pneumothorax, pulmonary interstitial emphysema, pneumoperitoneum, pneumopericardium, pneumomediastinum)
- Rate of any air leak syndrome associated with any intraventricular hemorrhage

All other adverse events meeting the following criteria of seriousness will be considered unexpected serious adverse events and must be documented in the AE-from of the eCRF.

*uSAE* (any adverse event that resulted in any of the following):

- Death
- Serious deterioration in the health of the subject that resulted in any of the following:
  - Life-threatening illness or injury,
  - Permanent impairment of a body structure or a body function,
  - Medical or surgical intervention to prevent life-threatening illness or injury or permanent impairment to a body structure or a body function.

To ensure that there is no higher risk of deaths caused by the intervention, this will be evaluated with the help of 95% confidence limits that will be presented for both treatment groups. A DMC manual will describe the details of the safety analysis and has to be approved by all DMC members before preparing the first DMC report.

### Frequency and plans for auditing trial conduct {23}

Monitoring for this study is provided by the Center for Pediatric Clinical Studies (CPCS) of the University Hospital Tuebingen. Central monitoring of the eCRF will also be provided by the CPCS, certified by ISO9001. Monitoring is employed primarily for the subjects’ safety, as well as for quality assurance of medical procedures. The centers will be visited by the monitor on a regular basis. In accordance with the laws on data protection, the investigator’s files, data collection forms, and original documents have to be made available to the monitor. The investigators will discuss the course of the study with the monitor in an appropriate manner. Trial institutions, facilities, laboratories, and all data (including raw data and eCRFs) must always be available for inspection by an authority.

### Plans for communicating important protocol amendments to relevant parties (e.g., trial participants, ethical committees) {25}

Any additions and changes made to the protocol have to be submitted to the responsible Ethics Committees for review. Changes to protocol procedures (amendments) require a specification of reasons and must be signed by an authorized person for the respective protocol; the amendments are then considered part of the protocol. Substantial changes, in particular with regard to patients’ health interests, require a new decision from the appropriate Ethics Committees.

### Dissemination plans {31a}

The final report for the study will be compiled by the coordinating investigators within a period of 360 days upon completion of the study and forwarded to the ethics committees. Furthermore, the coordinating investigators will submit the final report for publication as soon as possible.

#### Consent for publication

Not applicable

## Discussion

Nasal CPAP application with selective surfactant administration is currently the primary and recommended respiratory support in very low birth weight infants with developing or active RDS [1, 4]. CPAP has been proven to be at least as effective as primary intubation in the delivery room with regard to the outcome of BPD and significantly reduced the need for invasive mechanical ventilation in large randomized controlled trials [1, 6, 7]. However, reported CPAP failure rates in these trials are still high (46.0–51.2%) and CPAP failure was ultimately linked with the exposure to mechanical ventilation, an intervention clearly associated with the diagnosis of BPD [5, 14, 16]. PEEP levels used in these trials ranged from 4 to 8 cmH_2_O. The evidence for the decision to apply these specific PEEP levels of 4–8 cmH_2_O in very immature preterm infants is low but considered to be safe. From a pathophysiological perspective, early application of PEEP to the surfactant deficient lungs counteracts the collapsing properties, maintains FRC, and supports lung liquid clearance immediately after birth [17]. In an attempt to accelerate lung liquid clearance, sustained increases of continuous positive airway pressure (i.e., sustained lung inflation maneuvers (SLI)) have been proposed to effectively recruit gas exchange units after birth [18]. While this SLI strategy has been shown to reduce the need for intubation within the first 72 h of life in VLBWI, the results of the SAIL trial showed no difference in the rate of BPD, but an increased mortality in the SLI group discouraging the use of this aggressive recruitment technique [18, 19]. Effective lung liquid clearance is necessary to allow gas exchange, but the approach of SLI does not take into account the fact that interstitial liquid tends to re-accumulate in the pulmonary tissue over a longer period of time [20]. In addition, RDS caused by primary surfactant deficiency of the premature lungs predisposes atelectasis and consecutive impaired gas exchange. It is considered a longer lasting dynamic disease process and therefore may require prolonged and sufficient PEEP support [21]. Early surfactant treatment, in the recent years predominantly administered via less invasive application procedures or the INSURE technique in the neonatal population, further reduced the need of invasive ventilation, but unfortunately did not yield the desired reduction in the incidence of BPD in multicenter studies [7, 14]. Stepwise recruitment strategies (IN-REC-SUR-E – Intubate Recruit Surfactant Extubate) using invasive high frequency oscillatory ventilation, where FiO_2_ serves as surrogate marker of effective lung inflation, combined with surfactant administration are currently under investigation and have been shown to reduce oxygen requirements and the incidence of BPD in one randomized controlled trial and retrospective studies [15, 22, 23]. SLI maneuvers and the INRECSURE procedures aim to apply higher pressures over a short period of time indicating that the immature preterm lung may require higher continuous distending pressures than expected in the past. However, clinical trials assessing the application of early, prolonged and higher levels of non-invasive PEEP to avoid collapse of the neonatal lung during nasal CPAP are not available but may be an alternative or adjunctant to the abovementioned ventilation and treatment strategies. The hypothesis that early application of “higher” PEEP levels could be beneficial is supported by animal data where higher PEEP levels (8–12 cmH_2_O) were more effective than lower levels in terms of gas exchange [12, 13]. More recently, a dynamic increase of PEEP in preterm lambs (up to 14–20 cmH_2_O for 3 min) improved response to surfactant treatment and created a more uniform lung aeration, compared to positive pressure ventilation or SLI maneuvers [24]. Further evidence that higher pressure levels may decrease the need for invasive mechanical ventilation derives from studies using NIPPV as a mode of non-invasive respiratory support. This ventilation strategy augments CPAP by superimposing positive pressure inflation at different respiratory rates, imitating time-cycled pressure-controlled ventilation. Results of the actual meta-analyses showed a reduction of the need for intubation and reintubation compared to nasal CPAP when NIPPV was used as primary mode of ventilation or post extubation [25, 26]. It is unclear how NIPPV mediates its clinical benefits, but one possible explanation could be the increase in mean airway pressure during NIPPV [27].

Concerns that may have discouraged the further assessment of higher levels of PEEP in the neonatal population might derive from results of the COIN trial where infants in the CPAP group had a higher rate of pneumothoraces with the use of a PEEP of 8 cmH_2_O [1]. This association, however, has to be interpreted with caution. As the authors stated, the median CPAP level was 8 cmH_2_O for infants who developed a pneumothorax as well as for those in whom a pneumothorax did not occur. If the CPAP pressure would have been the cause for the development of pneumothorax, it is interesting that the incidence of air leaks was lower in the intubation group, where infants were exposed to higher peak and mean airway pressures as compared to the CPAP group. A more reasonable explanation of these findings might be the lower rate of infants treated with surfactant in the CPAP-group and the high FiO_2_ intervention threshold for intubation and surfactant administration (FiO_2_ > 0.6), since surfactant treatment has been proven to reduce the incidence of air leaks in preterm infants [21]. The SUPPORT and CURPAP study with a similar study design and similar PEEP levels of 5–8 cmH_2_O did not find any difference in the incidence of air leaks, but had a lower threshold (i.e., FiO_2_ > 0.4) for surfactant administration [6, 7]. To our surprise, no clinical trial since the introduction of nasal CPAP was conducted that assessed the efficacy of different PEEP levels during nasal CPAP treatment for RDS in the preterm population. Given the facts that the available strategies to avoid mechanical ventilation did not (yet) result in a reduction of BPD and are still associated with high treatment failure rates and that other promising strategies (i.e., cell based therapies) have to be critically evaluated before introduction into clinical practice, it seems conclusive to improve the effectiveness of the well-established CPAP therapy as the currently accepted gold standard of non-invasive respiratory support in VLBWI [28, 29]. Our hypothesis is that the early application of a higher (6–8 cmH_2_O) versus a lower (3–5 cmH_2_O) PEEP range will more effectively recruit and maintain lung volumes in preterm infants with GA 26 + 0–29 + 6 weeks during the first 120 h of life and further reduce the need for mechanical ventilation and respiratory failure. To avoid exposure to unnecessary high continuous distending pressures, we further decided to follow the approach of titrating the level of PEEP within the randomized range according to the fraction of inspired oxygen to maintain arterial oxygen saturation in the target SpO_2_ range.

There are potential limitations in our study design that we addressed in the pre-study meetings: (1) all participating centers use nasal CPAP and NIPPV as primary respiratory support and post extubation. Because settings (inspiratory time, peak inspiratory pressure and respiratory rate) during NIPPV significantly affect the applied mean airway pressure and gas exchange, this could potentially result in unexpected and undesired differences in the study outcomes if this strategy is used unevenly between the two treatment groups. To resolve this issue, we decided that settings and weaning during the study intervention must be equally applied to both treatment arms in terms of inspiratory times, respiratory rate, and peak inspiratory pressures, which will be monitored and documented during the study intervention. The preparation of a center-specific protocol for the use of NIPPV prior to study initiation is strongly recommended and communicated during the pre-study meeting. (2) Analysis of the pre-study surveillance protocol revealed that participating centers use different modes of surfactant administration (INSURE or less invasive surfactant administration) as well as different thresholds and indications (FiO_2,_ pCO_2_, work of breathing) for surfactant administration. However, participating centers follow local guidelines ensuring that the indications and the mode of surfactant application are used similar between the two study groups within each center. (3) One of the primary endpoints is defined as need for intubation and invasive mechanical ventilation > 1 h. Although we strongly recommend that infants should be intubated if FiO_2_ ≥ 0.5 for > 1 h and/or pCO_2_ ≥ 70 mmHg in two consecutive blood gas analysis > 2 h apart after the application of a maximum of 2 surfactant doses, some physicians may however withheld intubation for any reasons. Therefore, we have chosen the criteria FiO_2_ ≥ 0.5 for > 1 h and/or pCO_2_ ≥ 70 mmHg in two consecutive blood gas analysis > 2 h apart after the application of a maximum of 2 surfactant doses as fulfillment of the primary endpoint as well (treatment failure). The abovementioned indicators for intubation (FiO_2_, pCO_2_) will be documented on an hourly basis. (4) Some physicians may have reservations against very high or very low PEEP settings that may lead to protocol violations. During the study preparation process and the pre-study meetings, there was a high agreement between the participating centers for the use of the two defined PEEP ranges. The ventilator parameters PEEP and FiO_2_ are recorded on an hourly basis to confirm protocol compliance or detect violence regarding the intended PEEP settings, which will then be discussed with the study monitor and the responsible investigator at the study site as soon as possible.

## Trial status

The start of recruitment is expected to be May 01, 2020. The estimated date of completed recruitment is July 31, 2022. The protocol version number is 1.2. The date of the protocol version is December 04, 2019.

## Abbreviations

CPAP: Continuous positive airway pressure
PEEP: Positive end-expiratory pressure
FiO_2_: Fraction of inspired oxygen
VLBWI: Very low birth weight infants
RDS: Respiratory distress syndrome
BPD: Bronchopulmonary dysplasia
FRC: Functional residual capacity
GA: Gestational age
GCP: Good clinical practice
NIPPV: Nasal intermittent positive pressure ventilation
INSURE: Intubate surfactant extubate
LISA: Less invasive surfactant administration
CPCS: Center for pediatric clinical studies
DMC: Data monitoring committee
PSN: Patient screening number
ISF: Investigator site file
eCRF: Electronic case report form
eSAE: Expected serious adverse event
uSAE: Unexpected serious adverse event
SLI: Sustained lung inflation
INRECSURE: Intubate recruit surfactant extubate
